# EID3 Promotes Cancer Stem Cell-Like Phenotypes in Osteosarcoma through the Activation of PI3K-AKT Signaling Pathway

**DOI:** 10.1155/2022/5941562

**Published:** 2022-08-28

**Authors:** Yan Wang, Shiyong Luo, Yuxuan Wang, Shengbang Yang, Zhitong Huang, Xuejin Zhu, Shanghua Cai, Qifeng Guo, Weide Zhong, Sihong Liu

**Affiliations:** ^1^Department of Urology, Guangdong Key Laboratory of Clinical Molecular Medicine and Diagnostics, Guangzhou First People's Hospital, South China University of Technology, Guangzhou, Guangdong 510180, China; ^2^Department of Orthopedics, Guangzhou First People's Hospital, Guangzhou Guangdong 510182, China; ^3^Department of Orthopedics, The Second People's Hospital of Panyu, Guangzhou, Guangdong 510182, China

## Abstract

The aim of this study is to elucidate molecular mechanism by which E1A-like inhibitor of differentiation 3 (EID3) promotes cancer stem cell-like phenotypes in osteosarcoma. Overexpression of EID3 in osteosarcoma cells generated more spherical clones, enhanced the expression of stemness-associated genes, and promoted chemoresistance, invasion, and metastasis. Furthermore, osteosarcoma cells overexpressing EID3 had increased ability to grow in suspension as osteospheres with high expression of Sox2 and stem cell marker CD133. In addition, knockdown of EID3 reduced sphere formation and inhibited osteosarcoma cell migration and invasion. RNA sequencing and bioinformatics analysis revealed that PI3K-Akt signaling pathway and MAPK pathway­related genes were enriched in osteosarcoma cells with high expression of EID3. Taken together, EID3 promotes osteosarcoma, and EID3–PI3K-Akt axis is a potential therapeutic target for osteosarcoma treatment.

## 1. Introduction

Osteosarcoma is the main cause of tumor death in children and adolescents [1]. With the development of modern medical technology, radical surgery and neoadjuvant chemotherapy has significantly increased the 5-year survival rate to 60% [2, 3]. However, due to the resistance of tumor cells to chemotherapeutic drugs, the survival rate has reached a plateau [4]. The submachine system of chemotherapy resistance of osteosarcoma is not yet clear, and the existence of osteosarcoma stem cells (OSCs) is considered to be a major reason for chemotherapy resistance of osteosarcoma [5, 6]. Although the role of osteosarcoma stem cells in chemotherapy resistance has not been fully elucidated, evidence shows that tumor stem cells can inhibit apoptosis through a variety of mechanisms, such as high expression of special drug transporters and effective DNA repair in osteosarcoma cells, which is related to the ability of tumor stem cells to maintain tumorigenicity through self-renewal and differentiation [5–7]. Therefore, to find the molecular mechanism related to the osteosarcoma stem cells and develop new targets to enhance drug sensitivity of osteosarcoma is an urgent problem to improve the treatment of osteosarcoma [8].

Molecular genetic analysis showed that the inactivation of tumor suppressor Rb and p53 played an important role in the occurrence and development of human osteosarcoma [9]. In vivo studies have also shown that osteosarcoma can be induced by mutations of genes such as (MSC) and/or bone progenitor cells such as p53 and RB or abnormal signal transduction of Hedgehog and NOTCH in mesenchymal stem cells [9, 10]. Recent studies have shown that osteosarcoma contains OSCs responsible for tumorigenesis, growth, recurrence, and chemoresistance [11]. OSCs can maintain their stemness through self-renewal and differentiation [12]. The molecular mechanism of chemoresistance of osteosarcoma is not clear, but the existence of OSCs is considered to be a major reason for chemoresistance of osteosarcoma.

Based on the self-renewal characteristics of stem cells, several methods have been developed to identify and isolate OSCs [13]. Functional in vitro tests for the formation of tumor spheres under nonadhesive and serum-free conditions are usually used as an initial step in the enrichment of OSC-like cell populations. The in vivo verification of OSCs is to evaluate the tumorigenicity of low cell count by continuously transplanting isolated hypothetical OSC into immunocompromised mice [14]. At present, proteins that can be used as surface markers of osteosarcoma stem cells are CD133, CD117, and Stro1; especially, CD133 is widely used [15, 16].

E1A-like inhibitor of differentiation 3 (EID3) is the third member of the EID family [17]. EID3 is homologous to a region of EID1, binds to p300/CBP, and acts as an inhibitor of p300/CBP-dependent transcription by direct interaction with nuclear receptors SHP and SRC1 [17]. EID is associated with a variety of tumors, and tumors expressing EID have strong invasiveness and poor prognosis. As a member of EID family, EID3 can inhibit histone acetyl transfer of CBP/p300 enzyme activity, and different from EID1, EID3 is specifically highly expressed in the testis [18]. In general, EID3 degrades rapidly through ubiquitin-dependent protein degradation pathway at the end of the cell cycle [19, 20], but the inactivated pRb protein mutation will lead to the stability of EID3 protein, resulting in the inhibition of cell differentiation [17, 21]. In fact, it has been reported that colon cancer cells with high expression of EID3 are more resistant to radiotherapy and chemotherapy and promote the formation of tumor stem cells [22]. In human umbilical cord blood mesenchymal stem cells, EID3 is highly expressed, and EID3 expression decreases during induced differentiation into neural stem cells [23]. However, the role of EID3 in osteosarcoma has not been reported.

In this study, we investigated the expression and biological function of EID3 in osteosarcoma cells. We demonstrated that the mRNA and protein levels of EID3 significantly increased in osteosarcoma cells. We found that osteosarcoma cells overexpressing EID3 generated more osteospheres and promoted cell invasion and had high expression of Sox2 and the stem cell marker CD133. Furthermore, RNA sequencing and bioinformatics analysis revealed that EID3 regulated stemness by interacting with PI3K-Akt signaling pathway.

## 2. Materials and Methods

### 2.1. Cell Culture

The human fetal osteoblast cell line hFOB and the human osteosarcoma cell lines MG-63 and U-2 OS were purchased from the American Type Cell Culture Collection (ATCC, USA) and cultured in DMEM-F12 (Gibco, USA), DMEM (Gibco, USA), and McCoy's 5a MeMo (Gibco, USA) plus 100 units/mL penicillin, 100 mg/mL streptomycin, and 10% fetal bovine serum (FBS), respectively. The human osteosarcoma cell line MNNG/HOS cells were purchased from the Cell Bank of the China Science Academy (Shanghai, China) and cultured in RPMI 1640 plus 100 units/mL penicillin, 100 mg/mL streptomycin, and 10% FBS. The hMSCs were kindly provided by Dr. Caixia Wang (Guangzhou First People's Hospital, Guangzhou, China) and cultured in conditioned medium composed of DMEM, 1 mmol/L L-glutamine (Gibco Laboratories, USA), 1% penicillin-streptomycin (Invitrogen, USA), and 10% FBS. All cells were maintained at 37°C with 5% CO_2_ and 100% humidity except that the hFOB cells were maintained at 34°C.

### 2.2. Sphere Formation Assay

Cells were plated in serum-free medium DMEM/F12 supplemented with B27 (Thermo Fisher Scientific, USA), 10 ng/mL epithelial growth factor (EGF), and 10 ng/mL basic fibroblast growth factor (bFGF) (Peprotech, USA) in ultralow attachment 6-well plates (Corning, USA) at a concentration of 1.0 × 10^3^ cells/well. Cells were incubated for 10-14 days, and spheres were counted under microscope (Olympus, Japan).

### 2.3. Lentivirus Transduction

The EID3-expressing lentivirus vector LV5-EID3 was constructed by the insertion of a full-length EID3 cDNA into LV5 vector (GenePharma, Shanghai, China). The LV5-EID3 and LV5 control (LV5-NC) vectors were transfected into 293FT cells for packaging. Viral supernatants were collected after 48 h as previously described [24].

### 2.4. Quantitative Real-Time PCR

Total RNA was extracted using the RNeasy Plus Mini Kit, and the concentration and purity was determined using an ND-1000 spectrophotometer as previously reported [24]. Total RNA was prepared and detected. The primers are shown in Table 1.

### 2.5. Western Blotting

Total cellular proteins were extracted with protein lysis buffer. Lysates were centrifuged at 10,000*g* at 4°C for 10 min, and supernatants were collected. The concentrations of proteins were detected by BCA Protein Assay Reagent Kit (Thermo, USA). Cell lysates containing 40 *μ*g protein were separated on a 12% SDS-PAGE gel (Bio-rad, USA) and then transferred on polyvinylidene difluoride (PVDF) membranes (Millipore, USA). The membranes were blocked with 5% bovine serum albumin (BSA) for 1 h; incubated with primary antibodies for EID3, SOX2, and GAPDH; and then washed and incubated with horseradish peroxidase-conjugated secondary antibodies for 1 h at room temperature. Finally, membranes were developed using an enhanced chemiluminescent (ECL) kit. Quantification of bands was performed using ImageJ Software.

### 2.6. Cell Viability Assay

Cells were seeded in 96-well microplates at a density of 4,000 cells per well. Cells were treated with different concentrations of doxorubicin (DOX) for the indicated hours. Next, Cell Count Kit-8 (CCK-8) solution (10 *μ*L) was added to each well. Finally, cell viability was measured with a microtiter plate reader (Bio-Tek).

### 2.7. Transwell Assay

Cell invasion was evaluated by the Matrigel invasion assay with a Corning Invasion Chamber (8 *μ*m pore size) (Corning, USA) according to the manufacturer's instructions. 1 × 10^4^ cells were seeded into the upper chamber of each well in serum-free medium, and the bottom chambers were filled with DMEM containing 10% FBS as chemoattractant. Cells were seeded in 10 mm diameter transwell plates with polycarbonate filters. After incubation for 24 h, the noninvading cells were gently removed with a cotton swab. Invasive cells were fixed for 30 min in 4% formaldehyde and stained for 15 min with crystal violet, air dried, and photographed. The number of invading cells was counted in five evenly spaced fields using an inverted phase-contrast microscope.

### 2.8. Wound Healing Assay

Cell migration was assessed by wound healing assay. In brief, cells were seeded in six-well plates in DMEM supplemented with 10% FBS. A scratch was created using a 200 *μ*L tip and washed twice with serum-free medium. The migration was measured at 0 h and 24 h. Three images were taken per well, and data were analyzed using ImageJ software.

### 2.9. FACS Analysis

Osteosarcoma cells were collected and washed with 0.5 mL of phosphate-buffered saline (PBS). Cells were incubated with PE-conjugated anti-human CD133 antibody (Miltenyi Biotec) or respective isotype controls at 4°C for 30 minutes in the dark. After washing, the labelled cells were analyzed on flow cytometer (BD Bioscience, USA), and data were analyzed using FlowJo 10.2 software (FlowJo, USA).

### 2.10. EID3 Silencing by shRNA

MG-63 osteosarcoma cells were transfected with human EID3 shRNA (YSH-LV001-EID3 [shRNA1/2/3]; Ubigene, Guangdong, China). The shRNA2 sequence targeting EID3 corresponded to coding regions (5′-CTCGTACTGTGGAGAATATAT-3′, antisense 5′-GAGCATGACACCTCTTATATA-3′) of the EID3 gene. The EID3 knockdown stable cell lines were established by adding 5.0 *μ*g/mL puromycin in the complete medium for 48 h. Surviving cells were EID3 knockdown stable cells.

### 2.11. RNA Sequencing and Bioinformatics Analysis

Total RNA was extracted by RNeasy mini kit (Tianmo) for quality inspection using Agilent Bioanalyzer 2100 (Agilent technologies, Santa Clara, CA, US). The library was constructed on the cBOT of Illumina NovaSeq 6000 sequencer in accordance with the standard process and hybridization of the first sequencing primer. The RNA reads were then aligned to the reference sequences using the 2-pass mode of STAR_2.4.0b (default parameters) 55, and relative gene expression was quantified as transcript per million (TPM) using RSEM v1.2.17 (default parameters) 56. Isoform expression levels for each gene were summed to derive the TPM values. To remove genes with low expression values, the following steps were applied. First, TPM values < 1 were considered unreliable and substituted with zero. Second, TPM values were log2-transformed after adding a value of one. Third, genes expressed in <10% of all tumor groups were removed. There were a total of 118,949 genes. DEseq (version 1.14.0) was used to call differentially expressed genes (DEGs) in our samples. To define DEGs, we set up a stringent statistic cutoff of fold change (FC) of ≥2 and the false discovery rate (FDR) of <0.05. A total of 487 DEGs was identified between MG-63-EID3 and MG-63-Vector. H-cluster analysis was used to analyze the expression of DEGs, and functional enrichment was analyzed.

### 2.12. Statistical Analysis

The data are presented as the mean and the error bars. All analyses were performed using GraphPad Prism software (GraphPad Software, Inc.). Statistical significance was determined by an unpaired Student's *t*-test. *p* values of < 0.05 were considered statistically significant.

## 3. Results

### 3.1. EID3 Is Highly Expressed in Osteosarcoma Cells

**Figure 1:**
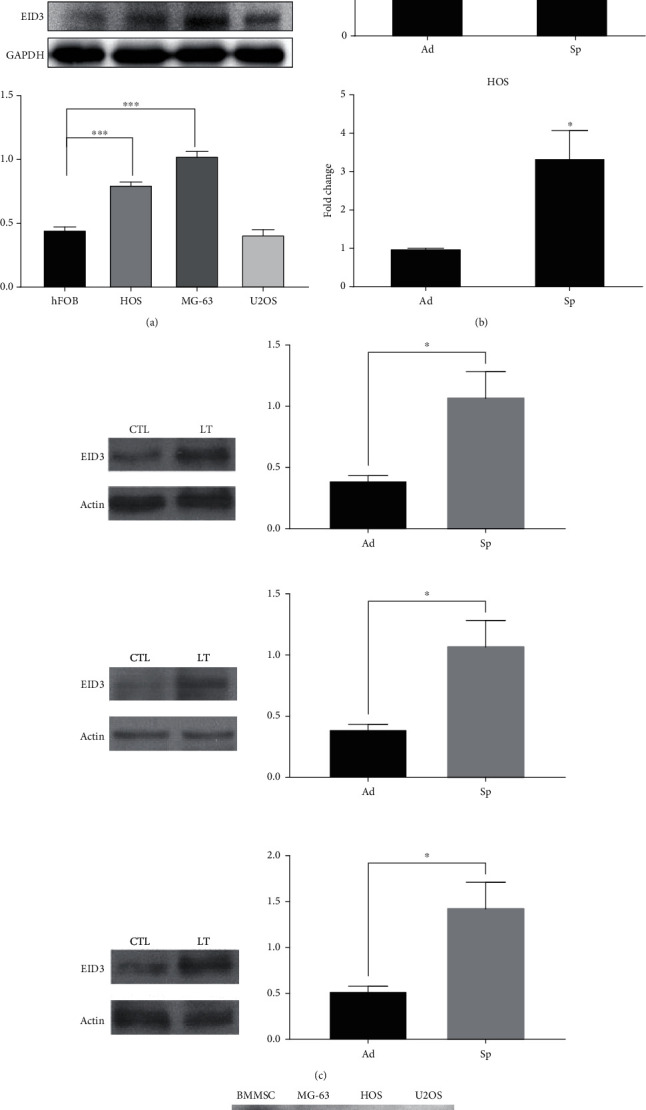
EID3 is highly expressed in osteosarcoma cell lines. (a) Western blot analysis of EID3 expression in osteosarcoma cells and osteoblast cell line. (b) Analysis of EID3 mRNA expression in different osteosarcoma cells. (c) Western blot analysis of EID3 expression in adhere cultured or sphere-cultured osteosarcoma cell lines. (d) Western blot analysis of EID3 expression in hMSC and osteosarcoma cell lines. All data are presented as mean ± SE (*n* = 3). ∗, *p* < 0.05; ∗∗, *p* < 0.01; ∗∗∗, *p* < 0.001.

We examined EID3 expression in three human osteosarcoma cell lines and found that EID3 protein was overexpressed in all three osteosarcoma cell lines compared to primary human osteoblasts cell line hFOB. As shown in Figure 1(a), we observed that the expression levels of EID3 in osteosarcoma MG-63 cells was higher than that of other osteosarcoma cells, including U2OS and HOS cells. We further determined the expression levels of EID3 in sphere-cultured and monolayer-cultured MG-63 cells [25, 26]. We found that EID3 expression was higher in the sarcospheres than in adherent cells (Figures 1(b) and 1(c)). In addition, EID3 expression was higher in bone mesenchymal stem cells (BMSCs) than in other osteosarcoma cells (Figure 1(d)).

### 3.2. EID3 Overexpression Enhances Stemness of Osteosarcoma Cells

**Figure 2:**
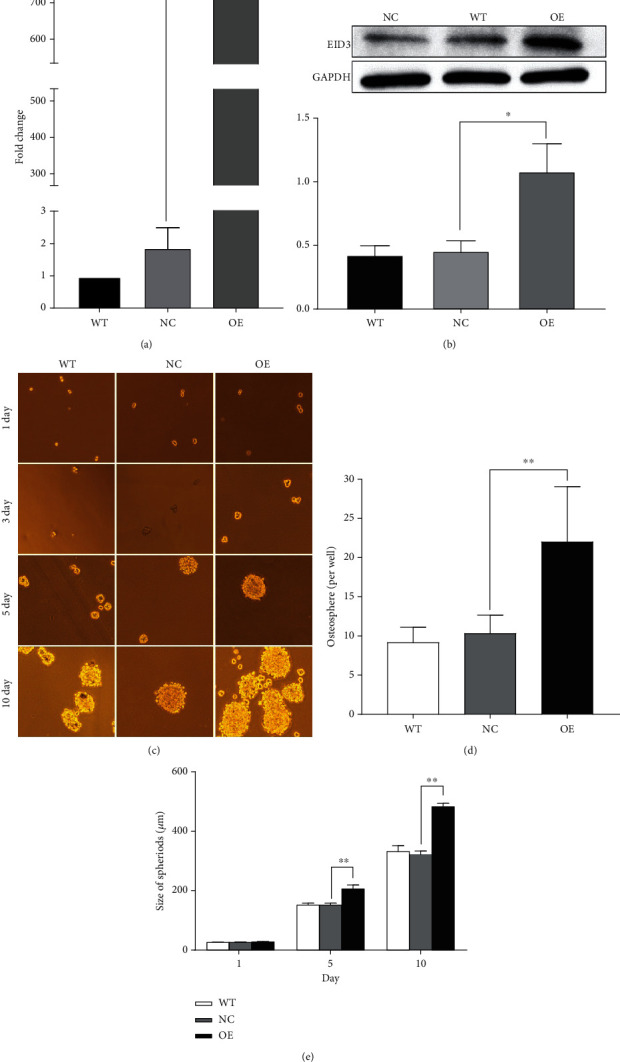
EID3 overexpression promotes stem-like properties of MG-63 cells. (a and b) EID3 protein and mRNA expression levels in MG-63 cells after transduction with LV5-EID3 or LV5 vector. (c) The spheroid-forming abilities of MG-63-EID3 and MG-63-Vector cells in tumorsphere culture. (d) Quantitation of the sphere-forming assay. (e) The diameter of data are shown as mean ± SD, *n* = 3. ∗∗, *p* < 0.01.

**Figure 3:**
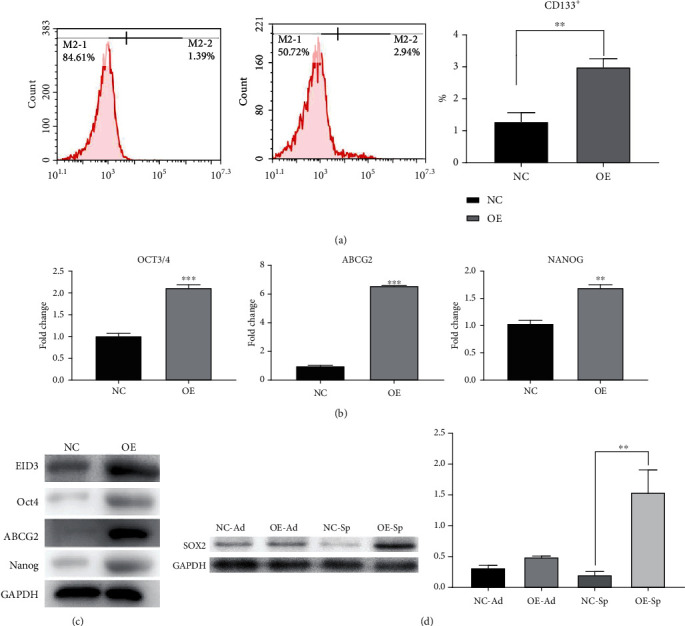
EID3 overexpression promotes stemness of MG-63 cells. (a) Cytometry analysis of CD133^+^ cells in MG-63-EID3 and MG-63-Vector cells. (b) Overexpression of EID3 upregulated mRNA levels of osteosarcoma stem cell markers OCT3/4, ABCG2, and NANOG. (c) Overexpression of EID3 upregulated protein levels of osteosarcoma stem cell markers OCT3/4, ABCG2, and NANOG. (d) Western blot analysis of protein levels of stem cell marker SOX2. All data are presented as mean ± SE (*n* = 3). ∗, *p* < 0.05; ∗∗, *p* < 0.01; ∗∗∗, *p* < 0.001.

EID3 plays an important role in tumor stem cells. To determine whether EID3 can enhances stemness of osteosarcoma cells, we generated EID3-overexpressing MG-63 cell line (MG-63-EID3) and control cell line (MG-63-Vector) (Figures 2(a) and 2(b)). Overexpression of EID3 increased osteosphere formation and dimension (Figures 2(c)–2(e)). Next, we assessed the proportion of CD133^+^ cells in MG-63-Vector and MG-63-EID3 cells. The results showed that overexpression of EID3 significantly increased the ration of CD133^+^ cells in MG-63 cells (Figure 3(a)). Three CSC-related genes including OCT3/4, ABCG2, and NANOG were overexpressed at mRNA and protein levels in MG-63-EID3 cell lines (Figures 3(b) and 3(c)). Moreover, SOX2 expression significantly increased in MG-63-EID3 cells compared to MG-63-Vector cells (Figure 3(d)). These data suggest that EID3 may facilitate the enrichment of stem cell-like osteosarcoma cells.

### 3.3. EID3 Promotes the Migration and Chemoresistance of Osteosarcoma Cells

**Figure 4:**
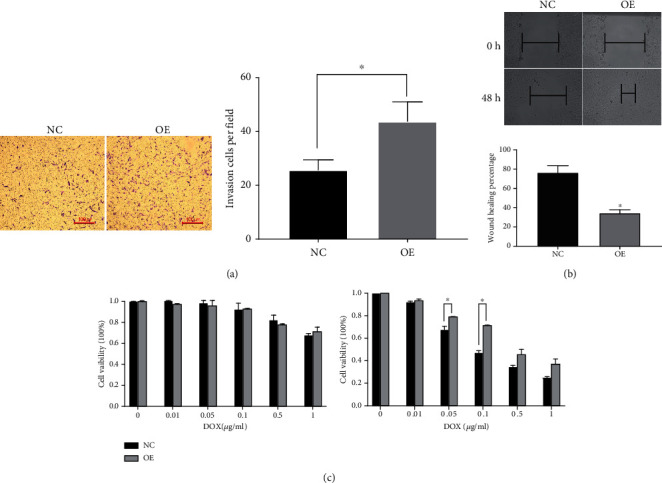
Overexpression of EID3 enhances invasion, migration, and chemoresistance of osteosarcoma cells. (a) Transwell assay of the invasion of MG-63-EID3 and MG-63-Vector cells. (b) Wound healing assay of the migration of MG-63-EID3 and MG-63-Vector cells. (c) Cells were treated with various concentrations of DOX for 48 h. Cell proliferation was measured by CCK-8 assay. Data are shown as mean ± SD, *n* = 3. ∗, *p* < 0.05; ∗∗, *p* < 0.01; ∗∗∗, *p* < 0.001.

Transwell assay showed that the number of invasive cells increased approximately 72% after infection with LV5-EID3 (MG-63-EID3) compared with MG-63 cells infected with the LV5 vector (MG-63-Vector) (*p* < 0.001) (Figure 4(a)). Wound healing assay showed that EID3 overexpression enhanced cell migration capability (Figure 4(b)). In addition, CCK-8 assay showed that osteosarcoma cancer-derived cells with EID3 overexpression exhibited higher viability after treatment with DOX than control cells (Figure 4(c)).

### 3.4. Knockdown of EID3 Attenuates Stemness, Invasion, and Chemoresistance of Osteosarcoma Cells

**Figure 5:**
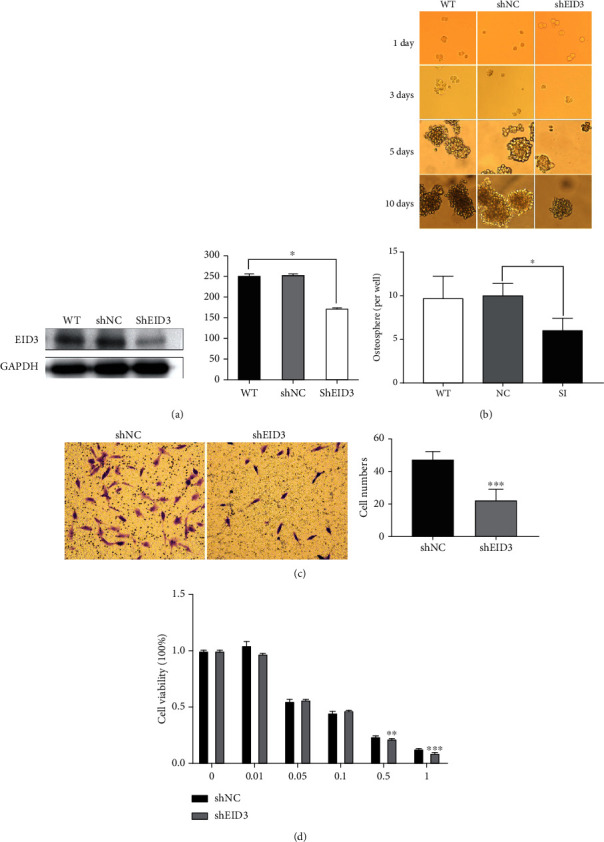
Knockdown of EID3 regulated spheroid forming, invasion, and chemoresistance of osteosarcoma cells. (a) Western blot analysis of EID3 in MG-63 cells transfected with EID3 shRNA or control shRNA for 48 h. (b) The spheroid-forming abilities of MG-63-shRNA and MG-63-shEID3 cells in tumorsphere culture. (c) Transwell assay of the invasion of MG-63-shRNA and MG-63-shEID3 cells. (d) Cells were treated with EID3 shRNA or control shRNA and DOX for 48 h. Cell proliferation was measured by CCK-8 assay. All data are presented as mean ± SE (*n* = 3). ∗, *p* < 0.05; ∗∗, *p* < 0.01; ∗∗∗, *p* < 0.001.

To further explore the function of EID3 in OSCs, we employed EID3-specific shRNA to knockdown EID3 in MG-63 cells (Figure 5(a)). Sphere-forming assay showed that EID3 shRNA-transduced MG-63 cells had reduced osteosphere formation (Figure 5(b)). Transwell assay showed that EID3 shRNA attenuated the invasion of MG-63 cells (Figure 5(c)). In addition, CCK-8 assay showed that EID3 shRNA decreased the viability of MG-63 cells after treatment with DOX (Figure 5(d)).

### 3.5. PI3K-Akt Signaling Is Required for EID3-Mediated Osteosarcoma Stemness

**Figure 6:**
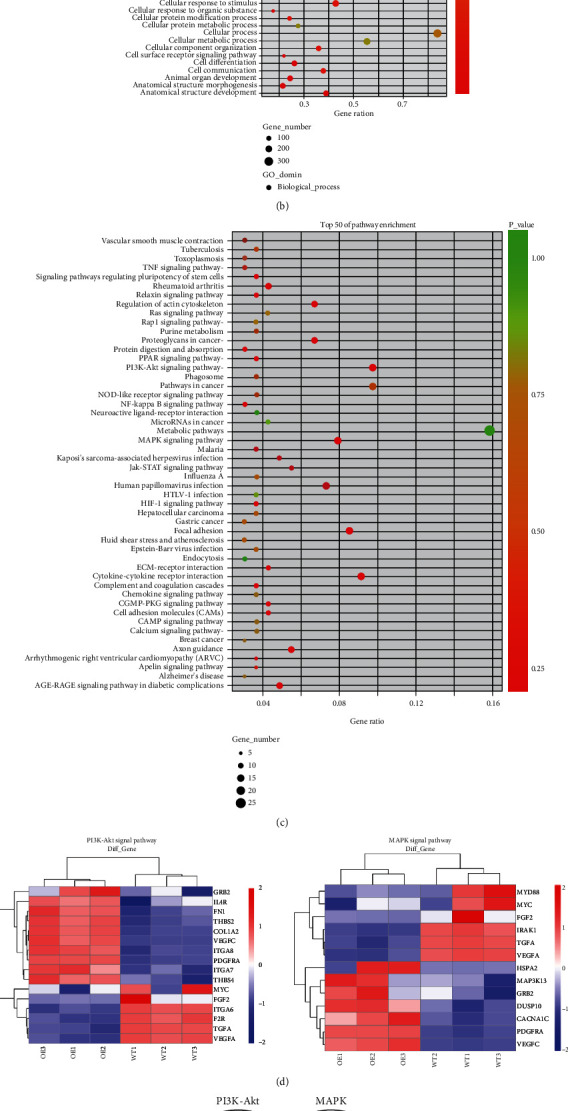
RNA sequencing and bioinformatics analysis. (a) The volcano plot showing mRNAs with differential expression between the two groups. Red and blue plots represent up- and downregulated genes, respectively. (b) The enriched GO annotation for DEGs between MG-63-EID3 cells and MG-63-Vector cells. (c) KEGG pathway enrichment analysis for DEGs between MG-63-EID3 cells and MG-63-Vector cells. (d) Profiling of differentially expressed PI3K-AKT and MAPK signaling pathway-related genes. (e) The overlapping genes differentially expressed between the two groups of cells. (f) PCR analysis of the genes of PI3K-AKT and MAPK pathways from the overlapping genes described in (e).

To shed light on the mechanism of EID3-induced osteosarcoma cancer stemness, transcriptome sequencing was performed to examine the effector genes. Genes that showed log2|fold change| ≥ 1.5 upregulation or downregulation in all the six paired samples was defined as DEGs or effector genes. A total of 111 DEGs were identified, 58 were upregulated, and 53 were downregulated (Figure 6(a)). Gene Ontology analysis showed that the biological processes of DEGs focused primarily on the regulation of cellular processes, multicellular organism development, and cellular response to stimulus. The Kyoto Encyclopedia of Genes and Genomes pathway analysis showed that DEGs were mainly involved in PI3K-Akt signaling pathway, MAPK signaling pathway, cytokine-cytokine receptor interaction, focal adhesion, and regulation of actin cytoskeleton (Figure 6(b)). Notably, our data revealed that EID3 overexpression could activate several signaling pathways that maintain self-renewal capacity, including PI3K-Akt, MAPK, cytokine, and Jak-Stat pathways (Figures 6(c) and 6(d)). Microarray analysis demonstrated that overexpression of EID3 upregulated the genes involved in PI3K-Akt signaling pathway, including GRB2, PDGFRA, VEGFC, IL4R, FN1, THBS2, ITGA7, ITGA8, and COL1A2 (Figure 6(e)). The upregulation of GRB2, PDGFRA, MYC, VEGFA, and VEGFC was further confirmed by PCR (Figure 6(f)).

**Figure 7:**
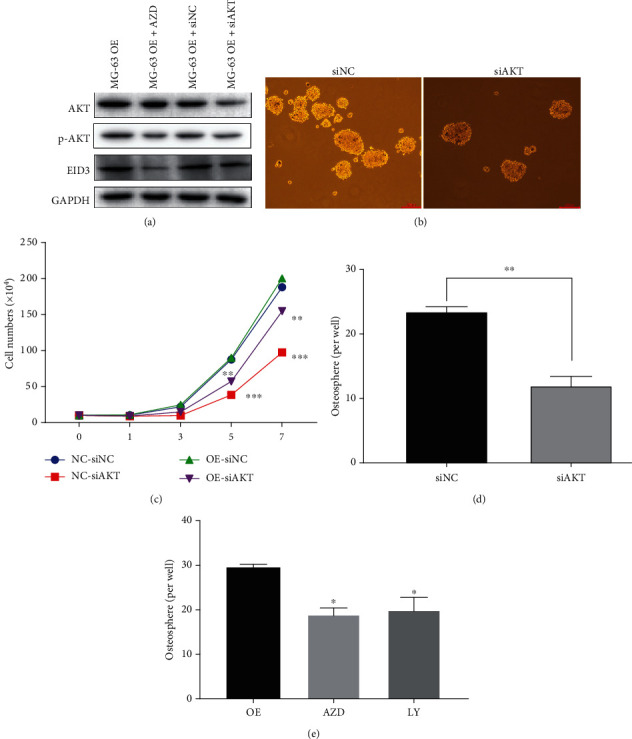
Involvement of PI3K-Akt pathway in EID3-mediated stemness of osteosarcoma cells. (a) Western blot analysis of Akt, p-Akt, and EID3 in MG-63-EID3 cells transfected with siRNA-Akt or Akt inhibitor AZD. (b) Cell proliferation was measured by CCK-8 assay. (c) Representative images of the spheres in MG-63-EID3 cells transfected with siRNA-Akt or control siRNA. Scale bar, 100 *μ*m. (d) Quantitation of sphere forming in MG-63-EID3 cells transfected with siRNA-Akt or control siRNA. (e) Quantitation of sphere forming in MG-63-EID3 cells treated with Akt inhibitor and PI3K inhibitor. AZD5363: AKT inhibitor. LY 294002: PI3K inhibitor. Data are shown as mean ± SD, *n* = 3. ∗∗, *p* < 0.01.

Next, we inhibited Akt by using Akt-specific siRNA or Akt special inhibitor in MG-63-EID3 cells (Figure 7(a)). The knockdown of AKT significantly inhibited the viability of MG-63-EID3 cells (Figure 7(b)). Moreover, Akt-specific siRNA decreased the number of spheres induced by EID3 overexpression (Figures 7(c) and 7(d)). Furthermore, Akt inhibitor (AZD5363) and PI3K inhibitor (LY294002) significantly decreased the number of spheres induced by EID3 overexpression (Figure 7(e)). Taken together, these findings indicate that PI3K-Akt signaling pathway contributes to EID3-induced osteosarcoma cancer stemness.

## 4. Discussion

Osteosarcoma is the most commonly diagnosed primary malignant bone tumor, with a peak in incidence occurring in the second decade of life. OSCs play important role in osteosarcoma. It is well recognized that EID3 represses transcription and inhibits cell differentiation [1]. In human umbilical cord blood mesenchymal stem cells, EID3 is highly expressed, and EID3 expression decreases during the induced differentiation into neural stem cells [23]. Recent studies have focused on the role of EID3 in tumorigenesis. It has been reported that colon cancer cells with high expression of EID3 participate in the inhibition of differentiation of colon cancer cells and the formation of tumor stem cells [22]. Our results showed that EID3 was highly expressed in MG-63, U2OS, and HOS osteosarcoma cell lines, especially in MG-63 cells. Furthermore, the stem cells in osteosarcoma cells were enriched by sphere culture, and the expression of EID3 was increased in these osteosphere cells. Tirino et al. showed that CD133, a membrane glycoprotein, may be a marker of CSCs in osteosarcoma [27]. CD133^+^ cells were identified in three osteosarcoma cell lines (Saos2, MG63, and U2OS). These results suggest that EID3 may be related to the stemness of osteosarcoma cells. Further in vivo studies based on animal models are needed to confirm the role of EID3 in the maintenance of the stemness of osteosarcoma cells.

To explore the role of EID3 in osteosarcoma, we overexpressed or deleted the expression of EID3 in osteosarcoma cells and proved that EID3 played an important role in maintaining the stemness of osteosarcoma cells based on sphere-forming assay, chemoresistance, and cell migration and invasion assay. EID3 overexpression not only improved stem cell phenotype but also enhanced the enrichment of CD133^+^ cells and the expression of stem cell-related markers OCT3/4, ABCG2, and NANOG in osteosarcoma cells [14].

Next, we used transcriptome sequencing to explore the mechanism by which EID3 regulates the stemness of osteosarcoma cells. The results showed that DEGs were mainly involved in PI3K-Akt signaling pathway, MAPK signaling pathway, cytokine-cytokine receptor interaction, and focal adhesion. Moreover, we found that overexpression of EID3 can lead to high expression of GRB2, PDGFRA, MYC, VEGFA, and VEGFC gene. EID3 interacts with CBP and p300 to inhibit gene transcription and cell differentiation in part via the inhibition of histone acetyltransferase (HAT) activity of p300. Whether EID3 maintains the stemness of osteosarcoma cells by upregulating the expression of GRB2 and activating PI3K-AKT pathway remains to be further explored.

## 5. Conclusions

In conclusion, our study demonstrates high expression of EID3 in osteosarcoma cells, especially in sphere-cultured osteosarcoma cells. EID3-overexpressing MG-63 cells exhibited significantly higher sphere-forming activity and higher levels of GRB2, PDGFRA, MYC, and VEGFA. These findings reveal the mechanism by which EID3 promotes the stemness of osteosarcoma cells and chemoresistance and provides new approach for targeted therapy for osteosarcoma patients.

## Figures and Tables

**Table 1:** 

Gene	Forward primer	Reverse primer
EID3	CGGTTTCTTGTTATGGCTTCTGATTTG	CAGGATGTTGCTTCCTTTTCTATTGC
POU5F1	ATGTGGTCCGAGTGTGGTTC	GGACAGGGGGAAAGGCTTC
ABCG2	CATCAACTTTCCGGGGGTGA	CACTGGTTGGTCGTCAGGAA
NANOG	ATGGTGTGACGCAGGGATG	TGCACCAGGTCTGAGTGTTC
GRB2	AAAGCTACTGCAGACGACGA	GCCTTGGCTCTGGGGATTTT
VEGFA	TCTGCTTTTAAGGCCCCTGTG	CTCAATTCCTTCCCCCAGCA
VEGFC	GCAGTTACGGTCTGTGTCCA	CGACTCCAAACTCCTTCCCC
PDGFRA	TAAAACCCACGGCCAGATCC	AGCTCCGTGTGCTTTCATCA
MYC	TCGGAAGGACTATCCTGCTG	GTGTGTTCGCCTCTTGACATT
GAPDH	CATGGGTGTGAACCATGAGAAGTA	CAGTAGAGGCAGGGATGATGTTCT

Gene expression levels were calculated using the 2-*ΔΔ*Ct method and normalized to the housekeeping gene glyceraldehyde-3-phosphate dehydrogenase (GAPDH).

## Data Availability

The data used to support the findings of this study are available from the corresponding author upon request.
